# Propensity Score Analysis of Second-Line Chemotherapy Outcome in Advanced Biliary Tract Cancer

**DOI:** 10.3390/jcm15062204

**Published:** 2026-03-13

**Authors:** Kijjakom Thanasombunsukh, Chaiyut Charoentum, Apichat Tantraworasin, Jiraporn Khorana

**Affiliations:** 1 Department of Internal Medicine, Lampang Hospital, Lampang 52000, Thailand; k_thanasombunsukh@hotmail.com; 2 Department of Biomedical Informatics and Clinical Epidemiology, Faculty of Medicine, Chiang Mai University, Chiang Mai 50200, Thailand; apichat.t@cmu.ac.th; 3 Division of Oncology, Department of Internal Medicine, Faculty of Medicine, Chiang Mai University, Chiang Mai 50200, Thailand; ccharoentum@gmail.com; 4 Department of Surgery, Faculty of Medicine, Chiang Mai University, Chiang Mai 50200, Thailand; 5 Clinical Surgical Research Center, Chiang Mai University, Chiang Mai 50200, Thailand

**Keywords:** biliary tract cancer, second-line chemotherapy, propensity score, inverse probability of treatment weighting, flexible parametric regression, restricted mean survival time

## Abstract

**Background/Objectives**: Several chemotherapeutic regimens and targeted therapies are currently established as standard second-line treatments for patients with advanced biliary tract cancer (BTC). However, evidence regarding the benefits of treatment after first-line therapy failure remains limited, particularly among Thai populations. This study aimed to explore the efficacy of second-line chemotherapy in patients with advanced BTC. **Methods**: We conducted a single-institution, retrospective study including patients with locally advanced or metastatic BTC who experienced disease progression following first-line treatment between January 2017 and December 2019. Overall survival (OS) was defined as the primary endpoint. The secondary endpoint was the restricted mean survival time (RMST). To minimize confounding, propensity scores were estimated and applied using inverse probability of treatment weighting (IPTW). **Results**: A total of 110 patients were included, of whom 69 (62%) received second-line chemotherapy in combination with best supportive care (2LCMT + BSC), while 41 (38%) received best supportive care (BSC) alone. The majority of cases were intrahepatic cholangiocarcinoma (73.9% and 70.7% in each group, respectively). The median OS was 5.3 months (95% CI 3.5–7.0) in the 2LCMT + BSC group and 1.0 months (95% CI 0.5–1.9) in the BSC-only group (unadjusted HR 0.40, 95% CI 0.26–0.59; *p* < 0.001). In IPTW-adjusted flexible parametric regression analysis, second-line chemotherapy was associated with a 53% reduction in the risk of death compared with BSC alone (*p* = 0.009). The restricted mean survival time (RMST) differences between groups at 3, 6, and 12 months were 1.3 months (95% CI 0.9–1.6; *p* < 0.001), 2.6 months (95% CI 1.9–3.3; *p* < 0.001), and 3.9 months (95% CI 2.7–5.1; *p* < 0.001), sequentially. **Conclusions**: These findings demonstrate that second-line chemotherapy provides a significant overall survival benefit compared with best supportive care alone in patients with advanced BTC.

## 1. Introduction

Biliary tract cancers (BTC) are tumors comprising the gallbladder, ampulla of Vater and cholangiocarcinoma (CCA), which is the second most common type of hepatobiliary cancer worldwide [1,2]. The incidence rate of CCA is low in the USA, Europe, and Australia but significantly higher in East Asia, including Japan, China, Korea, and Thailand [3,4].

In advanced-stage BTC, chemotherapy is the standard of care, with first-line treatment consisting of cisplatin–gemcitabine from the UK ABC-02 study [5]. Addition of the immune checkpoint inhibitors (ICI) Durvalumab and Pembrolizumab demonstrates overall survival (OS) improvements compared to cisplatin–gemcitabine alone [6,7]. After first-line therapy failure, there was no standard chemotherapy until the ABC-06 study revealed a survival benefit by comparing the use of 5-Fluorouracil–leucovorin–oxaliplatin (FOLFOX) with active symptom control (ASC) alone, which is recognized as the standard of care in second-line treatment [8]. The combination of nano-liposomal irinotecan (Nal-IRI) and 5-Fluorouracil (5FU) was also found to improve progression-free survival (PFS) compared to 5FU alone in a Korean phase 2b study [9]. Novel targeted agents are also recommended in patients with actionable genomic alterations. Nevertheless, treatment accessibility is still limited in some regions. Moreover, there is little available data regarding the benefit of treatment beyond first-line therapies, especially in Thai patients. The only retrospective, single-center study in Thailand was conducted to examine the efficacy of the FOLFOX regimen between 2014 and 2018; 19 patients were included [10]. The median OS was 6.2 months, and most of the patients (89%) achieved disease control. Therefore, the aim of this study was to evaluate the efficacy of second-line chemotherapy in advanced BTC.

## 2. Materials and Methods

### 2.1. Study Design

This retrospective cohort study was conducted at Lampang Hospital, Lampang, Thailand. The data were collected from the hospital’s cancer center registry. Patients who were diagnosed with advanced-stage or unresectable BTC and experienced disease progression after first-line treatment between January 2017 and December 2019 were included. The inclusion criteria were patients aged 18 years or older who received first-line treatment for advanced disease and had radiologic confirmation via computed tomography (CT) or magnetic resonance imaging (MRI) of disease progression after treatment, according to the Response Criteria in Solid Tumor (RECIST) version 1.1 [11]. The exclusion criteria were patients who had concomitant secondary cancer or received second-line treatment at other sites.

The study protocol was reviewed and approved by the Ethics Committee of Lampang Hospital, Lampang, Thailand (approval number: EC144/68). The committee waived the need for informed consent due to the retrospective design of the study. The study was conducted in accordance with the Declaration of Helsinki and received no external funding.

### 2.2. Data Collection

Patient data were collected from medical records, including demographics (age and gender), performance status according to the Eastern Cooperative Oncology Group (ECOG) scale, BTC subtype (CCA, gallbladder, or ampulla), tumor characteristics (pathology subtype and grading), previous systemic therapy, number of metastases, and CA19-9 and total bilirubin (TB) level after first-line disease progression. The neutrophil–lymphocyte ratio (NLR), recognized as an inflammatory and prognostic marker in various cancers, was also recorded [12,13].

The patients were divided into two groups: the first received second-line chemotherapy with best supportive care (2LCMT + BSC), and the second received best supportive care (BSC) alone. Treatment assignment was determined by patient preference following a thorough discussion with the attending physician.

The efficacy of second-line chemotherapy was evaluated using overall survival (OS), which was the primary outcome of the study.

All of the clinicopathological data of patients in the study were obtained and de-identified from the hospital cancer registry databases according to the Health Insurance Portability and Accountability Act (HIPAA) policy.

### 2.3. Statistical Analysis

The sample size was calculated based on power analysis for the exponential test of hazard ratio (HR) from a previous study by Moik et al. [14]. The adjusted hazard ratio of second-line chemotherapy for overall survival was 0.48. To obtain a statistical power of 80% and an alpha error of 5% (two-sided test), this study required at least 33 subjects in each group.

The primary endpoint was overall survival (OS), and the secondary endpoint was the restricted mean survival time (RMST). Categorical variables were reported as counts and percentages, while continuous variables were reported as means ± standard deviation (SD) or median with interquartile range (IQR) based on data distribution. Survival analysis was performed with a Kaplan–Meier curve and the log-rank test. Because the proportional hazards assumption was violated, survival analysis was conducted using flexible parametric models (FPM) with restricted cubic splines instead of Cox regression, reported as hazard ratio (HR). Propensity scores (PS) were calculated using a multivariable logistic regression model, and the inverse probability of treatment weighting (IPTW) method was applied to reduce effects from pre-treatment confounding factors. The propensity score was constructed using pre-treatment covariates, including age, gender, performance status, primary tumor origin, tumor histopathology, tumor grade, response to first-line chemotherapy regimen, presence of peritoneal metastases, total bilirubin level, tumor marker level, NLR, and number of metastatic sites. Standardized mean differences (SMD) were calculated to explain the imbalance between covariates. If residual imbalance persisted after IPTW, the double adjustment method was applied in the regression model to counteract the remaining confounders. The RMST in both 2LCMT + BSC and BSC arms with RMST differences were evaluated at fixed time points of 3, 6 and 12 months. Patients who survived at the evaluated time point were censored. A *p*-value below 0.05 was considered statistically significant, including FPM survival estimation and RMST differences. Stata Software version 16 (StataCorp LLC., College Station, TX, USA) was used for statistical analysis.

## 3. Results

### 3.1. Baseline Characteristics

**Figure 1:**
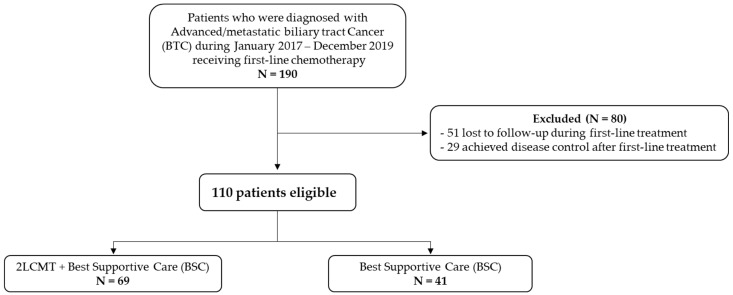
Flow diagram. (All of the patients in this cohort were dead; the time from treatment assignment to death ranged from 0.1 to 46 months).

A total of 110 patients were included in this study, of whom 69 (62%) received second-line chemotherapy with best supportive care (2LCMT + BSC) and 41 (38%) received best supportive care (BSC), as shown in Figure 1.

The subjects were predominantly male (63.8% and 61% of the 2LCMT + BSC and BSC groups, respectively), with a median age of 61.7 and 61.4 years. Intrahepatic cholangiocarcinoma (iCCA) was predominant, affecting 73.9% and 70.7% of patients. The most common pathologic subtype was adenocarcinoma (65.2% and 56.1%), and the most common frontline chemotherapy used was a cisplatin–gemcitabine regimen (68.1 and 70.7%). Baseline characteristics are shown in Table 1.

### 3.2. Survival Outcome

**Figure 2:**
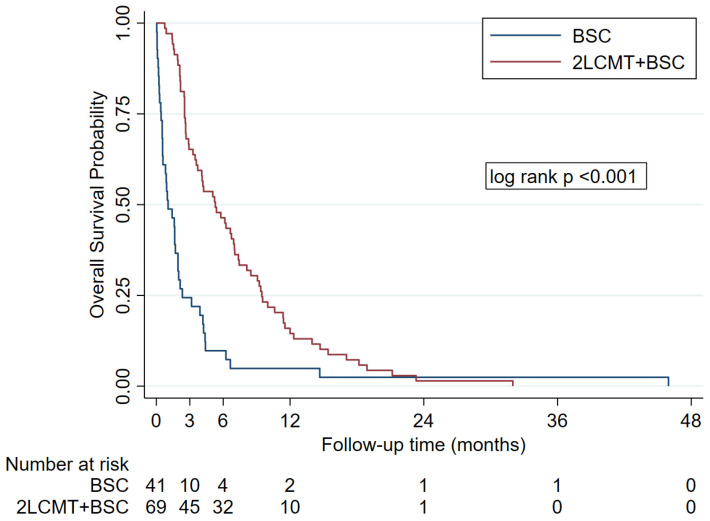
Kaplan–Meier curve for overall survival. (Abbreviations: BSC, best supportive care; 2LCMT + BSC, second-line chemotherapy with best supportive care).

Figure 2 shows the Kaplan–Meier curve for overall survival, with a median follow-up duration of 3.0 months (range: 0.1–46.0). The primary endpoint, median OS, was 5.3 months in the 2LCMT + BSC group (95% CI 3.5–7.0) compared to 1.0 months (95% CI 0.5–1.9) in the BSC group (unadjusted HR 0.40, 95% CI 0.26–0.59, *p* < 0.001). The OS rate at 3, 6 and 12 months was 65%, 46% and 14% in the 2LCMT + BSC group and 24%, 10% and 5% in the BSC group, respectively.

**Figure 3:**
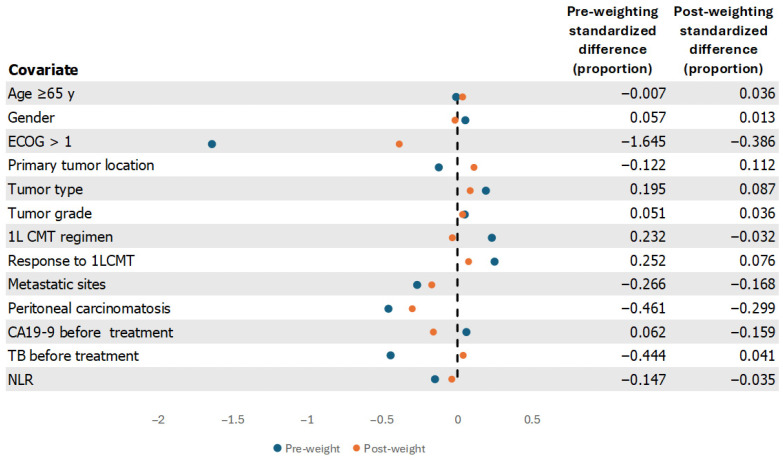
Standardized mean differences (SMD) before and after IPTW. (Abbreviations: ECOG, Eastern Cooperative Oncology Group; 1LCMT, first-line chemotherapy; CA19-9, carbohydrate antigen 19-9; TB, total bilirubin; NLR, neutrophil-lymphocyte ratio; IPTW, inverse probability of treatment weighting).

Potential prognostic factors associated with overall survival in the univariable analysis included good ECOG performance status, the number of metastatic sites, peritoneal carcinomatosis, total bilirubin > 1 mg/dL and a high neutrophil-to-lymphocyte ratio (NLR). Nevertheless, using multivariable analysis with a flexible parametric model revealed that >2 metastatic sites (HR 2.61; 95% CI 1.49–4.58; *p* = 0.001) and NLR ≥ 3 (HR 2.30; 95% CI 1.35–3.94; *p* = 0.002) were statistically significant prognostic factors, as shown in Table 2. We constructed propensity scores (PS) by applying logistic regression with baseline patients’ factors included in the study regardless of statistical significance. Inverse probability of treatment weighting (IPTW) analysis was implemented based on PS calculation. The covariate balance between groups, assessed by standardized mean differences (SMD), before and after IPTW, is depicted in Figure 3. Most of the covariates are well balanced; however, there are covariates with SMD exceeding the −0.1 and 0.1 boundary even after IPTW, including ECOG, primary tumor location, number of metastatic sites, presence of peritoneal carcinomatosis and CA19-9 level. Therefore, the double adjustment method with these factors was applied to enhance the efficacy of hazard ratio estimation. With IPTW-derived, multivariable analysis with flexible parametric regression, 2LCMT + BSC was associated with a 53% reduction in the risk of death compared to BSC alone (weighted HR 0.47; 95% CI 0.27–0.82; *p* = 0.009), as shown in Table 3.

The restricted mean survival time (RMST) was analyzed in unweighted samples at 3, 6 and 12 months, and was 2.7, 4.4 and 6.1 months in the 2LCMT + BSC compared to 1.4, 1.8 and 2.2 months in the BSC group, and the adjusted RMST differences were statistically significant (*p* < 0.001, Table 4). In the 2LCMT + BSC group, oxaliplatin-based chemotherapy (FOLFOX or CapeOX) was prescribed the most often (78.3%). The second-line chemotherapy regimen used in the study and the treatment response are summarized in Table 5.

## 4. Discussion

According to our study results, second-line chemotherapy with BSC resulted in improved survival outcomes for advanced BTC patients regardless of age, performance status (good/borderline), tumor location, previous first-line treatment and other factors. Independent prognostic factors included >2 tumor metastatic sites and a high NLR.

In general, the retrospective study design introduces limitations due to various confounders that could have contributed to the patients’ categorization into either treatment group. Inverse probability of treatment weighting (IPTW) analysis and propensity scores (PS) are statistical methods increasingly implemented to counteract these biases [15]. In brief, the propensity score gives the probability (range 0−1) of an individual subject being exposed to the intervention (treatment) according to their measured baseline characteristics, and IPTW analysis uses the propensity score to balance baseline patients in both exposed and unexposed groups by weighting each subject with the inverse probability of receiving actual treatment [16,17]. Standardized mean differences (SMD) are calculated to evaluate the balancing ability of the weighting method. In our study, most covariates’ SMDs after weighting were between −0.1 and 0.1 (in other words, the “absolute SMD” was less than 0.1), indicating good balance between groups. Nevertheless, some covariate imbalances persisted, even with IPTW adjustment. Among the remaining imbalanced covariates, including ECOG performance status, primary tumor location, number of metastatic sites, presence of peritoneal carcinomatosis, and CA19-9 level, primary tumor location was the least imbalanced (SMD 0.112). The remaining four factors have been identified as poor prognostic indicators in various retrospective studies and may have influenced patients’ decisions regarding whether to receive second-line treatment. Therefore, the double adjustment method with those factors was incorporated to reduce the residual confounding effect. Following the statistical adjustment, the HR estimation with weighted multivariable regression was favorable in the 2LCMT + BSC group, emphasizing the additional benefit of pre-exposure variable management (weighted HR 0.47; 95% CI 0.27–0.82; *p* = 0.009 vs. unweighted HR 0.40, 95% CI 0.26–0.59, *p* < 0.001). Compared to the previous propensity score analysis study in second-line BTC [14], our study showed a consistent reduction in the risk of death with both weighted (HR 0.47; 95% CI 0.27–0.82; *p* = 0.009 vs. 0.40, 95% CI 0.17–0.95, *p* = 0.037) and unweighted regression (HR 0.40, 95% CI 0.26–0.59, *p* < 0.001 vs. 0.36, 95% CI 0.20–0.64, *p* = 0.001). Nonetheless, cautious cross-study interpretation is advised, as both studies had different populations and variables contributing to the propensity score analysis.

Most oncology trials implement a time-to-event analysis to evaluate treatment efficacy, for which proportional hazards (PH) regression, such as Cox regression, is often used. However, if the PH assumption is not met, the HR estimation may be less appropriate. The restricted mean survival time (RMST), defined as the area under the curve of the survival graph up to a specified time point, could be used as an alternative [18]. The RMST represents the average survival duration during a follow-up period, providing a clinically interpretable result of overall survival outcome compared to the medians in traditional PH regression [19,20]. Our study showed that the magnitude of RMST difference between the 2LCMT + BSC and BSC groups was numerically and statistically consistent with the median OS improvement indicated by traditional analysis. To the best of our knowledge, no prior retrospective study in the advanced BTC, second-line chemotherapy setting has used the RMST to represent survival outcomes.

The randomized controlled trial (RCT) is currently recognized as the best study design for comparing the benefits of two treatment modalities. Nevertheless, as biliary tract cancers are generally rare, prospective RCTs may not be feasible and would need to be conducted across multiple institutions. According to the ABC-06 study [8], a landmark second-line chemotherapy trial that randomized advanced BTC patients between FOLFOX plus ASC versus ASC alone, the survival benefit was marginally improved, with a median OS of 6.2 months (95% CI 5.4–7.6) compared to 5.3 months (95% CI 4.1–5.8). However, the ASC arm showed longer survival than expected following patient selection, as most of the participants in the clinical trial had to meet inclusion/exclusion criteria. In our study, the 2LCMT + BSC group showed a greater magnitude of survival benefit compared to the ABC-06 patients (HR 0.40, *p* < 0.001 vs. HR 0.69, *p* = 0.031). Still, the median OS was numerically lower in both groups (5.3 vs. 6.2 months in 2LCMT and 1.0 vs. 5.3 months in BSC).

It may be difficult to directly compare our results with other real-world evidence, as most research has been conducted in single-arm studies evaluating patients receiving second-line treatment only [10,21,22,23,24]. To compare the treatment efficacy with the previous Thai single-center study, the median OS difference in the treatment arm was modest (5.3 vs. 6.2 months), which can reasonably be expected [10]. Our survival result was also slightly lower than that in the multi-center cohort in Italy and France (5.3 vs. 6.6 vs. 6.7 months, respectively) [22,23]. However, compared with the findings from a large retrospective cohort in the United States [24], the median OS in our study was considerably inferior to that in the second-line chemotherapy arm (5.3 vs. 10.9 months), suggesting differences in tumor biology and patient populations, as well as the influence of other undiscovered factors.

With advances in molecular profiling, potential targetable genetic alterations have been discovered, including IDH1 mutation, FGFR2 fusion, HER2 amplification, BRAF mutation, NTRK fusion, RET fusion, KRAS G12C mutation and MMR deficiency [25]. Recent targeted therapy trials in phase 1/2 showed promising activity, resulting in accelerated approval and implementation in various international guidelines as novel second-line treatments in the United States and Europe [26,27]. For instance, Ivosidenib in IDH1 mutation [28], Pemigatinib and Futibatinib in FGFR fusion [29,30], Trastuzumab Deruxtecan and Zanidatamab in HER2 positive [31,32], Dabrafenib/Trametinib in BRAF V600E mutant [33], Entrectinib and Larotrectinib in NTRK fusion [34,35], Selpercatinib in RET fusion [36], Adagrasib in KRAS G12C mutation [37] and Pembrolizumab in MMR-deficient tumors [38,39]. However, efficacy and accessibility in different regions must be studied in future clinical research.

The strengths of our study include providing real-world evidence of the benefit of second-line chemotherapy in patients with advanced BTC, which is considered a rare type of tumor, and representing one of the largest cohorts in Thailand to date. The statistical methods, including propensity score (PS) analysis with weighting, were applied to overcome study design limitations. Moreover, survival outcomes were estimated using the traditional median overall survival complemented by the restricted mean survival time (RMST), offering more flexible and clinically interpretable results regardless of statistical assumptions (PH or non-PH). Nevertheless, this study had some limitations. As it was conducted at a single institution, the patients’ characteristics may differ from those treated at other centers. Therefore, the generalizability of the treatment outcomes to other regions should be interpreted with caution. The retrospective design may have introduced selection bias. Although propensity score and IPTW methods were used to balance possible pre-treatment factors, these approaches can only adjust the measurable variables; therefore, unknown confounders may have affected the outcome. Additionally, the heterogeneity of second-line chemotherapy regimens used in this study may have influenced the overall magnitude of benefit. However, this impact is likely to be modest, as the majority of patients in the treatment group (78%) received the FOLFOX regimen, which is considered the current standard of care, and the observed overall survival benefit was comparable to that previously reported in randomized and real-world studies.

## 5. Conclusions

Second-line chemotherapy provides additional survival benefits in patients with advanced biliary tract cancer compared to best supportive care alone. Despite the limitations of this study’s retrospective design, the implementation of statistical methods, including propensity scores with inverse probability of treatment weighting (IPTW) analysis, further emphasizes the magnitude of survival benefit. Future prospective studies, including evaluations of novel therapy or combination chemotherapy–targeted therapy in second-line settings, are warranted.

## Figures and Tables

**Table 1:** Patients’ baseline characteristics.

Baseline Characteristics	2LCMT + BSC (*n* = 69)	BSC (*n* = 41)	*p*-Value ^€^
Age in years			
Mean ± SD*	61.7 ± 9.4	61.4 ± 10.9	0.856
Age < 65 y (N, %)	44 (63.8)	26 (63.4)	0.970
Age ≥ 65 y (N, %)	25 (36.2)	15 (36.6)	
Gender, N (%)			
Female	25 (36.2)	16 (39.0)	0.770
Male	44 (63.8)	25 (61.0)	
ECOG Performance status, N (%)			
0–1	62 (89.9)	11 (26.8)	<0.001
2	6 (8.7)	12 (29.3)	
>2	1 (1.4)	18 (43.9)	
Diagnosis, N (%)			
Intrahepatic CCA	51 (73.9)	29 (70.7)	0.870
Extrahepatic CCA	10 (14.5)	5 (12.2)	
Gallbladder cancer	7 (10.1)	6 (14.6)	
Ampullary cancer	1 (1.5)	1 (2.5)	
Tumor Histology, N (%)			
Adenocarcinoma	45 (65.2)	23 (56.1)	0.555
Other type	4 (5.8)	2 (4.9)	
Unknown	20 (29.0)	16 (39.0)	
Tumor grade, N (%)			
Well differentiated (WD)	13 (18.9)	5 (12.2)	0.663
Moderately differentiated (MD)	8 (11.6)	7 (17.0)	
Poorly differentiated (PD*)	9 (13.0)	4 (9.8)	
Unknown	39 (56.5)	25 (61.0)	
1LCMT, N (%)			
Cisplatin + Gemcitabine	47 (68.1)	29 (70.7)	0.056
Cisplatin + 5FU	7 (10.1)	9 (22.0)	
Others	15 (21.8)	3 (7.3)	
Best response to 1LCMT, N (%)			
Partial response (PR)	16 (23.2)	4 (9.8)	0.182
Stable of disease (SD)	23 (33.3)	14 (34.2)	
Progression of disease (PD)	30 (43.5)	23 (56.0)	
Number of metastatic sites, N (%)			
1–2	29 (42.0)	12 (29.3)	0.181
>2	40 (58.0)	29 (70.7)	
Peritoneal carcinomatosis, N (%)			
Yes	17 (24.6)	19 (46.3)	0.019
No	52 (75.4)	22 (53.7)	
CA19-9 before treatment, N (%)			
Median (IQR)	77.1	6310	0.007
	(8.3–2996.0)	(198.7–84,943.5)	
≤400 IU/mL	16 (23.2)	7 (17.1)	0.098
>400 IU/mL	10 (14.5)	13 (31.7)	
Unknown	43 (62.3)	21 (51.2)	
Total Bilirubin before treatment, N (%)			
Median (IQR)	0.5 (0.4–1.1)	1.0 (0.6–2.8)	<0.001
≤1 mg/dL	50 (72.5)	21 (51.2)	0.024
>1 mg/dL	19 (27.5)	20 (48.8)	
Neutrophil-lymphocyte ratio (NLR)			
Median (IQR)	3.8 (2.6–6.1)	5.5 (2.8–9.8)	0.078
NLR < 3	25 (36.2)	12 (29.3)	0.455
NLR ≥ 3	44 (63.8)	29 (70.7)	

Abbreviations: BSC, best supportive care; 2LCMT + BSC, second-line chemotherapy with best supportive care; SD*, standard deviation; ECOG, Eastern Cooperative Oncology Group; CCA, cholangiocarcinoma; WD, well differentiated; MD, moderately differentiated; PD*, poorly differentiated; 5FU, 5-fluorouracil; 1LCMT, first-line chemotherapy; CA19-9, carbohydrate antigen 19-9; PR, partial response; SD, stable of disease; PD, progression of disease; NLR, neutrophil-lymphocyte ratio; IQR, interquartile range. ^€^ *p*-values were calculated based on Student’s *t*-test, Chi-square or Mann–Whitney U test.

**Table 2:** Univariable and multivariable analysis with flexible parametric model (FPM) and prognostic factors for overall survival.

Prognostic Factor	Univariable			Multivariable		
	HR	95% CI	*p*-Value	HR	95% CI	*p*-Value
Treatment group BSC 2LCMT + BSC	Ref. 0.40	0.26–0.59	<0.001	Ref. 0.34	0.18–0.64	0.001
Age <65 y ≥65 y	Ref. 0.84	0.57–1.24	0.382	Ref. 0.66	0.38–1.15	0.142
Gender						
Female	Ref.			Ref.		
Male	1.02	0.69–1.50	0.924	1.29	0.80–2.11	0.297
ECOG performance status						
0–1	Ref.			Ref.		
>1	2.88	1.90–4.36	<0.001	2.02	0.99–4.10	0.052
Primary tumor location						
Intrahepatic CCA	Ref.			Ref.		
Extrahepatic CCA	0.72	0.41–1.28	0.266	1.34	0.66–2.70	0.411
Gallbladder cancer	1.05	0.58–1.89	0.863	1.83	0.86–3.89	0.115
Ampullary cancer	0.49	0.12–1.98	0.315	0.88	0.18–4.38	0.88
Tumor type						
Unknown	Ref.			Ref.		
Adenocarcinoma	1.12	0.73–1.70	0.6	1.28	0.72–2.29	0.397
Others	1.04	0.44–2.48	0.923	1.35	0.47–3.82	0.574
Tumor grade						
Unknown	Ref.			Ref.		
Well differentiated	0.99	0.58–1.69	0.981	1.1	0.56–2.15	0.782
Moderately Differentiated	0.9	0.51–1.59	0.724	0.84	0.39–1.80	0.662
Poorly differentiated	0.96	0.53–1.74	0.89	0.9	0.39–2.09	0.807
1LCMT						
Cisplatin-Gemcitabine	Ref.			Ref.		
Cisplatin/5FU	0.85	0.49–1.46	0.556	0.76	0.40–1.46	0.418
Others	0.82	0.49–1.38	0.458	1.07	0.54–2.12	0.834
Best response to 1LCMT						
PD	Ref.			Ref.		
SD/PR	0.73	0.50–1.07	0.111	0.79	0.50–1.24	0.303
Number of metastatic sites						
≤2	Ref.			Ref.		
>2	2.24	1.47–3.39	<0.001	2.61	1.49–4.58	0.001
Peritoneal Carcinomatosis						
No	Ref.			Ref.		
Yes	1.96	1.28–2.99	0.002	1.32	0.80–2.18	0.274
CA19-9 before treatment						
≤400 IU/mL	Ref.			Ref.		
>400 IU/mL	1.15	0.65–2.06	0.628	0.86	0.44–1.68	0.656
Unknown	1.39	0.86–2.24	0.179	1.63	0.89–2.96	0.111
Bilirubin before treatment						
TB ≤ 1 mg/dL	Ref.			Ref.		
TB > 1 mg/dL	1.8	1.21–2.67	0.003	1.5	0.87–2.60	0.147
Neutrophil-lymphocyte ratio						
NLR < 3	Ref.			Ref.		
NLR ≥ 3	1.9	1.26–2.85	0.002	2.3	1.35–3.94	0.002

Abbreviations: BSC, best supportive care; 2LCMT + BSC, second-line chemotherapy with best supportive care; ECOG, Eastern Cooperative Oncology Group; CCA, cholangiocarcinoma; 1LCMT, first-line chemotherapy; PR, partial response; SD, stable of disease; PD, progression of disease; CA19-9, carbohydrate antigen 19-9; TB, total bilirubin; NLR, neutrophil-lymphocyte ratio; Ref, reference.

**Table 3:** Univariable and multivariable analysis with IPTW-derived, flexible parametric model (FPM) and double adjustment using imbalance covariates.

Prognostic Factor	Univariable			Multivariable		
	HR	95% CI	*p*-Value	HR	95% CI	*p*-Value
Treatment group						
BSC	Ref.			Ref.		
2LCMT + BSC	0.62	0.33–1.17	0.138	0.47	0.27–0.82	0.009
ECOG performance status						
0–1	Ref.			Ref.		
>1	1.89	1.11–3.20	0.019	2.34	1.36–4.02	0.002
Primary tumor location						
Intrahepatic CCA	Ref.			Ref.		
Extrahepatic CCA	0.53	0.17–1.60	0.259	0.57	0.14–2.37	0.44
Gallbladder cancer	0.82	0.48–1.42	0.488	1.43	0.77–2.64	0.257
Ampullary cancer	0.54	0.24–1.23	0.144	0.79	0.41–1.50	0.474
Number of metastatic sites						
≤2	Ref.			Ref.		
>2	2.78	1.59–4.86	<0.001	2.78	1.26–6.10	0.011
Peritoneal Carcinomatosis						
No	Ref.			Ref.		
Yes	1.87	1.15–3.02	0.011	1.03	0.56–1.92	0.912
CA19-9 before treatment						
≤400 IU/mL	Ref.			Ref.		
>400 IU/mL	1	0.58–1.70	0.993	1.03	0.54–1.95	0.928
Unknown	1.31	0.81–2.12	0.262	1.59	0.92–2.75	0.095

Abbreviations: IPTW, inverse probability of treatment weighting; BSC, best supportive care; 2LCMT + BSC, second-line chemotherapy with best supportive care; ECOG, Eastern Cooperative Oncology Group; CCA, cholangiocarcinoma; CA19-9, carbohydrate antigen 19-9; Ref, reference.

**Table 4:** Restricted mean survival time (RMST) in the unweighted population.

	2LCMT + BSC		BSC				
Follow-Up Time (Months)	RMST		RMST		RMST Differences		*p*-Value
	Mean (Months)	95% CI	Mean (Months)	95% CI	Mean (Months)	95% CI	
3	2.7	2.5–2.8	1.4	1.1–1.7	1.3	0.9–1.6	<0.001
6	4.4	4.0–4.9	1.8	1.3–2.4	2.6	1.9–3.3	<0.001
12	6.1	5.2–7.0	2.2	1.3–3.0	3.9	2.7–5.1	<0.001

Abbreviations: BSC, Best supportive care; 2LCMT + BSC, Second-line chemotherapy with best supportive care; RMST, Restricted mean survival time.

**Table 5:** Second-line chemotherapy (2LCMT) regimen and treatment response.

Regimen	Patients, N (%)	Median Number of Cycles (Range)	Best Response, N (%)		
			PR	SD	PD ^€^
FOLFOX/CapeOX	54 (78.3)	5 (1–12)	3 (5.6)	14 (25.9)	37 (68.5)
Carboplatin plus 5-FU	6 (8.7)	2 (1–6)	0	1 (16.7)	5 (83.3)
Gemcitabine	3 (4.4)	4 (1–8)	0	1 (33.3)	2 (66.7)
Capecitabine	1 (1.4)	8	0	1 (100)	0
Others	5 (7.2)	6 (3–8)	0	2(40)	3 (60)

Abbreviations: FOLFOX, Fluorouracil/Leucovorin/Oxaliplatin; CapeOX, Capecitabine/Oxaliplatin; 5FU, 5-fluouracil; LV, Leucovorin; PR, partial response; SD, stable of disease; PD, progression of disease. ^€^ Clinical and/or radiological progression of disease.

## Data Availability

The original contributions presented in this study are included in the article. Further inquiries can be directed to the corresponding author.

## Abbreviations

BTC	Biliary tract cancer
CCA	Cholangiocarcinoma
iCCA	Intrahepatic cholangiocarcinoma
eCCA	Extrahepatic cholangiocarcinoma
GB	Gallbladder
FOLFOX	Fluorouracil/Leucovorin/Oxaliplatin
CapeOX	Capecitabine/Oxaliplatin
5FU	5-fluouracil
ICI	Immune checkpoint inhibitor
AE	Adverse event
CA19-9	Carbohydrate Antigen 19-9
NLR	Neutrophil-lymphocyte ratio
CT	Computed tomography
MRI	Magnetic resonance imaging
RECIST	Response Criteria in Solid Tumor
1LCMT	First-line chemotherapy
2LCMT	Second-line chemotherapy
BSC	Best supportive care
ASC	Active symptom control
SD*	Standard deviation
PR	Partial response
SD	Stable of disease
PD	Progression of disease
OS	Overall survival
FPM	Flexible parametric model
PS	Propensity score
IPTW	Inverse probability of treatment weighting
SMD	Standardized mean difference
RMST	Restricted mean survival time
PH	Proportional hazard
IDH1	Isocitrate dehydrogenase 1
FGFR	Fibroblast growth factor receptor
HER2	Human epidermal growth receptor 2
BRAF	V-Raf murine sarcoma viral oncogene homolog B
NTRK	Neurotrophic tyrosine receptor kinase
RET	Rearranged during transfection
KRAS	Kirsten rat sarcoma virus oncogene homolog
MMR	Mismatch repair
